# Human isoprenoid synthase enzymes as therapeutic targets

**DOI:** 10.3389/fchem.2014.00050

**Published:** 2014-07-22

**Authors:** Jaeok Park, Alexios N. Matralis, Albert M. Berghuis, Youla S. Tsantrizos

**Affiliations:** ^1^Department of Biochemistry, McGill UniversityMontreal, QC, Canada; ^2^Department of Chemistry, McGill UniversityMontreal, QC, Canada; ^3^Department of Microbiology and Immunology, McGill UniversityMontreal, QC, Canada

**Keywords:** isoprenoids, mevalonate pathway, farnesyl pyrophosphate, geranylgeranyl pyrophosphate, squalene

## Abstract

In the human body, the complex biochemical network known as the mevalonate pathway is responsible for the biosynthesis of all isoprenoids, which consists of a vast array of metabolites that are vital for proper cellular functions. Two key isoprenoids, farnesyl pyrophosphate (FPP) and geranylgeranyl pyrophosphate (GGPP) are responsible for the post-translational prenylation of small GTP-binding proteins, and serve as the biosynthetic precursors to numerous other biomolecules. The down-stream metabolite of FPP and GGPP is squalene, the precursor to steroids, bile acids, lipoproteins, and vitamin D. In the past, interest in prenyl synthase inhibitors focused mainly on the role of the FPP in lytic bone diseases. More recently pre-clinical and clinical studies have strongly implicated high levels of protein prenylation in a plethora of human diseases, including non-skeletal cancers, the progression of neurodegenerative diseases and cardiovascular diseases. In this review, we focus mainly on the potential therapeutic value of down-regulating the biosynthesis of FPP, GGPP, and squalene. We summarize the most recent drug discovery efforts and the structural data available that support the current on-going studies.

## Introduction

**Figure 1:**
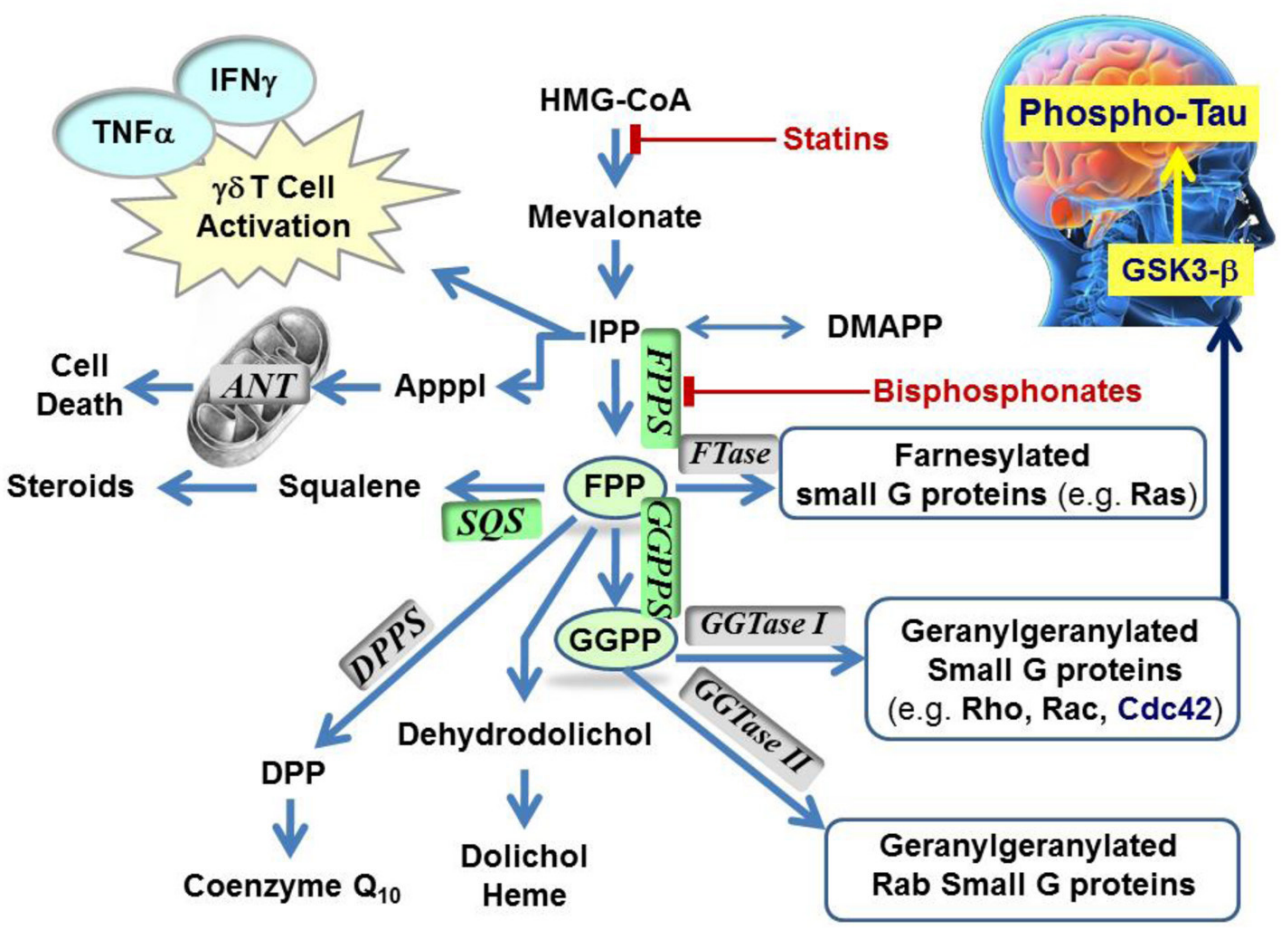
**Schematic representation of the mevalonate pathway, indicating the major biochemical effects of isoprenoids**. Classes of current drugs that modulate the function of key enzymes include Statins and Bisphosphonates (indicated 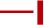). FPPS, GGPS, and SQS enzymes are highlighted in green color boxes; other key enzymes are highlighted in gray color boxes.

The mevalonate pathway is responsible for biosynthesis of all mammalian isoprenoids (Figure 1). These metabolites are essential for the post-translational prenylation of small GTP-binding proteins and serve as precursors to numerous other biomolecules, including cholesterol (which is essential for the integrity of cell membranes and is the precursor to all mammalian steroids, bile acids, lipoproteins, and vitamin D), haem A and ubiquinone (which are involved in electron transport), dolichol (essential for glycoprotein synthesis), and isopentenyladenine (found in tRNAs) (Goldstein and Brown, 1990).

**Figure 2:**
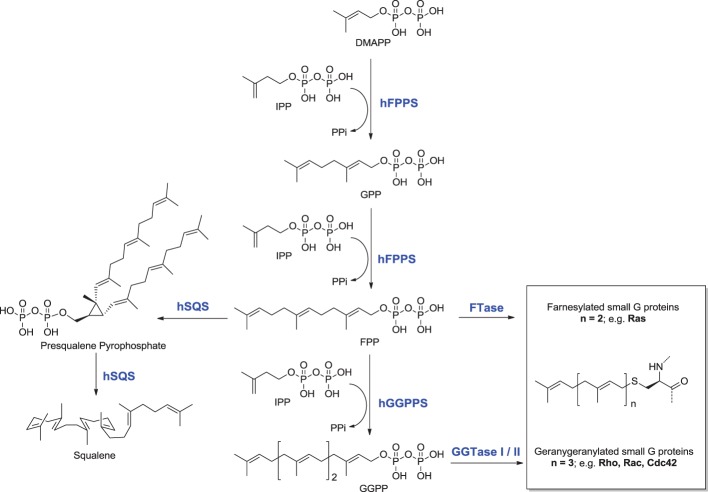
**Structures and sequence of steps involved in the biosynthesis of key isoprenoids**.

The rate-limiting step of the mevalonate pathway is catalyzed by hydroxymethylglutaryl coenzyme A reductase (HMC-CoA), leading to the formation of mevalonic acid. This metabolite is the immediate precursor of the 5-carbon isoprenoid monomer unit, isopentenyl pyrophosphate (IPP), and its isomer dimethylallyl pyrophosphate (DMAPP; Figure 2). Numerous reports have been written about HMG-CoA reductase as a therapeutic target and the value of the statin family of inhibitors in the treatment of atherosclerosis (Endo, 1992). Therefore, this account will focus mainly on the three down-stream prenyl synthase enzymes, the human farnesyl pyrophosphate synthase (hFPPS), geranylgeranyl pyrophosphate synthase (hGGPPS), and squalene synthase (hSQS). Based on pre-clinical and clinical data, the potential value of these enzymes in drug discovery will be discussed. The current state of the art in the design of inhibitors and the structural investigations that provide insight into the mechanisms of action of these inhibitors will also be described.

The first branching point of the mevalonate pathway is occupied by farnesyl pyrophosphate synthase (FPPS), the enzyme responsible for the catalytic elongation of DMAPP first to geranyl pyrophosphate (GPP) and then to FPP *via* the successive condensation of two IPP units (Figure 2). FPP is the substrate of geranylgeranyl pyrophosphate synthase (GGPPS), which catalyzes the down-stream extension of the C-15 isoprenoid (FPP) to the C-20 geranylgeranyl pyrophosphate (GGPP; Figure 2). Post-translational modification of proteins with either FPP or GGPP is estimated to constitute ~2% of the total mammalian proteome (Nguyen et al., 2009). Both FPP and GGPP are essential for the prenylation of small GTP-binding proteins (GTPases), a family of signaling proteins that are fundamentally important for cell survival (Takai et al., 2001). Covalent attachment of the FPP or GGPP lipid tail to a highly conserved cysteine residue at the CAAX box motif near/or at the C-terminal of a GTPase protein (where A is an aliphatic amino acid), confers membrane localization of this protein, promotes specific interactions with other proteins and plays a crucial role in controlling intracellular trafficking. Farnesylation is predominant when X is a methionine, serine, cysteine, or a glutamine residue, whereas geranylgeranylation usually occurs when X is leucine or isoleucine.

The known farnesylated proteins include members of the Ras superfamily of GTPases (e.g., H-Ras, K-Ras, N-Ras) (Kho et al., 2004), the precursor proteins of the nuclear lamin A (Young et al., 2005; Chang et al., 2012), and members of the DnaJ chaperone proteins (Kampinga and Craig, 2010; Stark et al., 2014). Geranylgeranylated GTPases include the Rho family of proteins, such as RhoA/B/C, Rac-1, and Cdc42 (Figure 1). Interestingly, the substrate specificity of the transferase enzymes which catalyze the prenylation step, FTase, and GGTase I and II (Figure 1) is not absolutely stringent and cross-prenylation has been observed (Rowell et al., 1997; Whyte et al., 1997; Yokoyama et al., 1997; Kho et al., 2004; Rowinsky, 2006). This redundancy mechanism has been blamed for the failure of FTase inhibitors (e.g., tipifarnib) to demonstrate significant clinical efficacy in the intended treatments of pancreatic (90% *K-Ras* mutations), lung, and colon carcinomas (~30% *K-Ras* mutations) (Lerner et al., 1997; Sparano et al., 2009).

## Overview of the human FPPS, GGPPS, and SQS as therapeutic targets

### Human FPPS

For over 40 years, drug discovery efforts focused mainly on the ability of hFPPS inhibitors to block osteoclast-mediated bone resorption; a number of excellent review articles have been written specifically on this topic (Dunford et al., 2001; Dunford, 2010; Ebetino et al., 2011; Russell, 2011). Currently, nitrogen-containing bisphosphonate (N-BP) inhibitors of hFPPS are used extensively to treat bone diseases, including osteoporosis, tumor-induced hypercalcemia, Paget's disease, and osteolytic cancer metastases (Fournier et al., 2010).

Chemically, N-BPs are stable analogs of naturally occurring pyrophosphates (diphosphates), such as inorganic pyrophosphate. At physiological pH, the bisphosphonate moiety is assumed to be ionized to the trianion (based on a pKa for the 3rd ionization of ~7.0) and further dissociates to the *tetra* anion in the presence of the enzyme's three co-factor Mg^2+^ ions. The structure of the current N-BP drugs features a Cα-hydroxyl substituent, which modulates the pKa of the two phosphonates and maximizes the affinity of these compounds for the bone mineral hydroxyapatite (Marma et al., 2007; Jahnke and Henry, 2010). N-BPs bind so avidly to bone that their half-life (in bone) can be months to several years, depending on the type of drug used and the disease affecting the rate of bone turnover (Grey et al., 2009; Frost et al., 2012). In chronic diseases (e.g., osteoporosis), concerns that prolonged use of high-dose N-BPs can cause side effects, such as osteonecrosis of the jaw (ONJ) and atypical femoral fractures (Rizzoli et al., 2008), have led to the recommendation by physicians of a “drug holiday.” However, this treatment can lead to uncertainty with respect to the type of drug and dose to be used, as well as the duration of treatment for different patients (Eastell et al., 2011). Although ONJ is uncommon in patients treated for osteoporosis, it is a long-lasting disorder that can occur in patients treated for bone cancer metastasis with high doses of intravenous bisphosphonates (Hoff et al., 2008; Dimopoulos et al., 2009; Ripamonti et al., 2009). The systemic half-life of current N-BPs is extremely low; for example, after i.v. administration of zoledronic acid (**1a)**, 50% of the dose gets trapped in the bone mineral and the rest is rapidly cleared by the kidneys. In fact, the dose-limiting toxicity of N-BP drugs, is usually based on nephrotoxicity (Skerjanec et al., 2003; Weiss et al., 2008). In spite of these limitations, N-BP drugs improve the quality of life for patients with lytic bone diseases; ~1 out of every 2 post-menopausal women suffers from osteoporosis and ~70% of all breast, prostate and multiple myeloma patients will develop bone metastases at some stage of their diseases. Currently, it is estimated that 400,000 patients/year in the USA alone suffer from lytic bone diseases due to cancer metastasis (Bagi, 2005; Coleman, 2006).

In addition to the skeletal benefits of N-BPs, recent pre-clinical studies have shown that they can suppress the proliferation of various types of cancer cells, including prostate (Iguchi et al., 2010; Mani et al., 2012), breast (Räikkönen et al., 2010; Dedes et al., 2012), and colorectal (Notarnicola et al., 2004) cancers, human glioblastoma (Cimini et al., 2011), and multiple myeloma (MM). Higher expression of hFPPS has been observed in human prostate cancer tissues (as compared to controls), suggesting an association between prenylation and disease progression (Todenhöfer et al., 2013). In the case of myeloma, a recent report of whole genome sequencing of tumors from 38 MM patients demonstrated that exactly 50% of these patients harbored either K-Ras or N-Ras coding mutations, underscoring the importance of these oncogenes in MM (Chapman et al., 2011). Clinical investigations have also provided evidence that some N-BP drugs (e.g., **1a**) improve the survival of patients with MM *via* mechanisms that are both related, as well as unrelated to the skeletal benefits (Morgan et al., 2010, 2012). Similar results have been reported for patients with premenopausal breast cancer (Gnant et al., 2009), although these findings seem to be more controversial (Coleman et al., 2011). In spite of all the preliminary evidence, the limited half-life of N-BPs in plasma (i.e., the fast elimination from the blood circulation) and negligible distribution to non-skeletal tissues compromise clinical validation of these compounds as true antineoplastic agents, and consequently, the clinical validation of hFPPS as a therapeutic target for non-skeletal malignancies.

To date, the observed N-BP-mediated anti-neoplastic effects have been attributed to multiple mechanisms relating to a plethora of biochemical processes that hFPPS controls (Figure 1). These mechanisms include the direct down-regulation of prenylated/activated mutated Ras proteins, thus inhibiting growth and/or survival of malignant cells. Inhibition of hFPPS also impacts the up-stream levels of isoprenoids in the mevalonate pathway leading to numerous cellular changes (Figure 1). For example, intracellular accumulation of the substrate IPP leads to accumulation of an ATP derivative, known as ApppI, which inhibits the mitochondrial adenine nucleotide translocase (ANT), inducing cell apoptosis (Mönkkönen et al., 2006; Mitrofan et al., 2009). IPP is also a natural antigen that directly stimulates γδ T cells caring the Vγ2Vδ 2 T cell receptors and is strongly implicated in the human innate immune response against tumors (Morita et al., 2007). Short hairpin RNA-mediated knockdown of hFPPS in hematopoietic and non-hematopoietic tumor cell lines has been shown to activate Vγ2Vδ 2 T cells and induce IFN-γ secretion (Figure 1) (Li et al., 2009; Wang et al., 2011). Immunostimulation and increased Vγ9Vδ 2 T cell-mediated cytotoxicity has been observed in animal models of human breast cancer after treatment with N-BPs, suggesting an adjuvant role for N-BPs in cancer chemotherapy (Benzaïd et al., 2012). Evidence for the stimulation of Vγ2Vδ 2-bearing T cells by N-BPs has also been observed in MM patients treated with palmidronic acid (**4**) (Kunzmann et al., 1999) and prostate cancer patients treated with zoledronic acid (**1a**) (Naoe et al., 2010); in the latter case, the observed T cell effects coincided with reduction in serum prostate-specific antigen (PSA), providing further evidence of an antitumor immune response *in vivo* (Naoe et al., 2010). It is noteworthy that activation of γδ T cells *in vitro* has been reported to correlate specifically with inhibition of hFPPS and has not been observed with structurally related N-BPs that target other downstream prenyl synthase enzymes, such as hGGPPS, hSQS or decaprenyl pyrophosphate synthase (hDPPS) (Zhang et al., 2010).

## Isoprenoids FPPS/GGPPS and neurodegeneration—the current hypothesis

In the early 1990s, a series of reports from Sparks and co-workers initiated a hypothesis linking the mevalonate pathway to the Alzheimer's disease (AD) (Sparks et al., 1990; Sparks, 1997). Evidence that dysregulation of the mevalonate pathway is involved in the progression of neurodegeneration and that high levels of plasma cholesterol may be among the main vascular risk factors of AD has been reported (Hoffman et al., 1997; Marchant et al., 2013). Although not a universal finding (Wood et al., 2014a), treatment of middle-aged individuals for hypercholesterolemia with statins has been shown to confer some level of neuroprotection against late-life development of AD (Jick et al., 2000; Wolozin et al., 2007; Silva et al., 2013; Wood et al., 2014b). Additionally, statin treatment has been shown to reduce the concentration of phosphorylated tau protein in the cerebrospinal fluid of the human brain (Riekse et al., 2006). One of the early pathological features of common AD is the progressive accumulation of ribbon-like intraneuronal tangles made of polymer of phosphorylated tau (P-Tau) protein, a key structural component of the cytoskeleton of neurons. These findings are consistent with the quasi-absence of cortical neurofibrillary tangles in autopsy-confirmed cognitively intact subjects who had used statins for several years as opposed to non-users (Li et al., 2007). Interestingly, although statins can cause a significant reduction in phosphorylated tau levels in the cerebrospinal fluid of patients treated for hypercholesterolemia, they do not seem to affect β-amyloid levels (Riekse et al., 2006).

Higher concentrations of both FPP and GGPP in the brain tissues of aged mice vs. younger mice have also been observed (Hooff et al., 2012). Up-regulation of hFPPS and hGGPPS in the brain of Alzheimer's patients has been reported (Eckert et al., 2009; Hooff et al., 2010). Furthermore, autopsy reports have indicated that the levels of FPP/GGPP are significantly elevated in gray and white matter of AD human brains, suggesting a disease-specific role for hFPPS and hGGPPS. Isoprenoids can presumably modulate Phospho-tau (P-Tau) levels in the human brain via the metabolic cascade from FPP→GGPP→Cdc42→GSK3-β (Figure 1) (Sayas et al., 1999; Ohm et al., 2003). The GSK3-β kinase is (at least in part) responsible for high levels of tau phosphorylation (P-Tau) in neurons (Hooper et al., 2008). Current research data indicate that decades prior to the onset of clinical dementia, the accumulation of P-Tau protein seems to trigger the formation of tangles, which progressively lead to neuronal cell death. Therefore, the biochemical association between dysregulation of isoprenoid biosynthesis and AD suggests that reducing the P-Tau burden in the AD brain by inhibiting either hFPPS or hGGPPS may decelerate (*or stop*) the progression of this disease. However, there is a considerable debate surrounding this hypothesis and its validation requires the discovery of potent and selective hFPPS and/or hGGPPS inhibitors with good biopharmaceutical properties that include significant permeability across the blood-brain barrier.

### Human GGPPS

The geranylgeranylated GTPase proteins (e.g., Rho, Ram, Rac, and Cdc42) play an indispensable role in signal transduction cascades of cell growth, differentiation, and survival. Although not identical, the pleiotropic biochemical consequences of hGGPPS inhibition parallel those of hFPPS inhibition. Consequently, numerous *in vitro* and *ex vivo* studies have suggested hGGPPS as a therapeutic target. For example, decrease in Rho kinase responses (as a consequence of statin treatment) has been implicated in increased production of endothelium-derived nitric oxide (NO) (Rikitake and Liao, 2005). Endothelial dysfunction is characterized as the decreased synthesis, release, and/or activity of endothelial-derived NO and is believed to be a strong predictor of cardiovascular diseases. Therefore, the regulation of NO by Rho may be an important mechanism underlying the cardiovascular protective effect of statins. Geranylgeranylated GTPases are also implicated in oncogenesis (Sorrentino et al., 2014). Inhibition of hGGPPS has been reported to decrease the migration/metastasis of highly invasive breast cancer cells (Dudakovic et al., 2011), induce autophagy in prostate cancer (Wasko et al., 2011) and play a role in the survival of glioma cells (Yu et al., 2014). Unfortunately, a clinically validated inhibitor of hGGPPS has not yet been identified, limiting the validation of this enzyme as a therapeutic target.

### Human SQS

Squalene Synthase (SQS) is a microsomal enzyme catalyzing the formation of squalene, the first committed step of the sterol branch and cholesterol biosynthesis in the mevalonate pathway (Figure 2). SQS can play a unique role in directing the flow of the metabolite FPP to either the sterol or the non-sterol branch of the pathway and due to this strategic location it is considered an attractive target for antihyperlipidemic and antiatherosclerotic therapies (Tansey and Shechter, 2001; Menys and Durrington, 2003; Do et al., 2009).

Despite the fact that today statins are the first line of treatment against hypercholesterolemia (competitive HMG-CoA reductase inhibitors), these drugs are often considered insufficient in preventing cardiovascular diseases (Gilad and Lampl, 1999; Huynh et al., 2002). Chronic inhibition of HMG-CoA reductase can lead to decreased levels of numerous non-sterol metabolites that are essential for cell viability (Levy and Kohlhaas, 2006). Furthermore, recent clinical studies suggest that the use of high doses of statins may lead to an increased risk of developing type II diabetes. Although a potential mechanism for this phenomenon is currently lacking, it has been suggested that statin-induced myopathy may be associated with the development of muscle insulin resistance (Mallinson et al., 2009). Nonetheless, given the clinical benefits of statins in patients with moderate or high cardiovascular risk factors, or existing cardiovascular diseases, discontinuation of treatment with statins was not recommended (Sattar et al., 2010; Preiss et al., 2011). The discovery of hSQS inhibitors as potential antihyperlipidemic agents will provide an alternative mechanism of action in treating hypercholesterolemia and may allow the limitations of statins to be overcome.

### Inhibitors of hFPPS

The clinically validated N-BP inhibitors of hFPPS, can be divided into two structural sub-classes, the aromatic and aliphatic bisphosphonates. The former includes zoledronic acid (**1a**), risedronic acid (**2a**), and minodronic acid (**3**), whereas the latter includes pamidronic acid (**4**), alendronic acid (**5**), and ibandronic acid (**6**). Under physiological conditions, the side chain nitrogen of N-BPs is presumed to be protonated and participate in bifurcated hydrogen bond interactions with the side chain hydroxyl moiety of Thr 201 and the carbonyl oxygen of Lys 200. These interactions mimic those formed with the putative allylic carbocation intermediate in the hFPPS catalytic cycle (Martin et al., 1999). These interactions are also critical for the potency of N-BPs in inhibiting hFPPS; for example, the phenyl analog of **2b** is 280-fold less potent than the pyridine derivative risedronic acid (**2a**) (Dunford et al., 2008).

**Figure:**
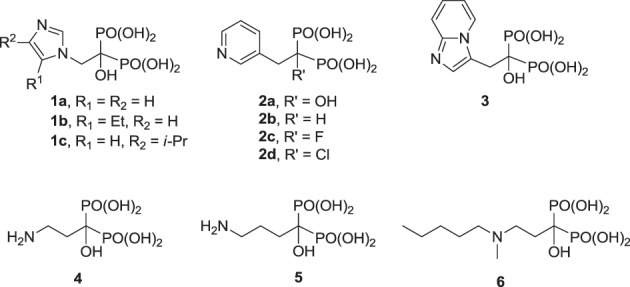


Previous efforts toward improving the clinical usefulness of N-BPs focused on increasing the oral bioavailability, cell-membrane permeability, and distribution to non-skeletal tissues. In principle, all of these biopharmaceutical properties depend on the physicochemical properties of the compounds. Pro-drug approaches, such as masking the bisphosphonate pharmacophore as a tetra ester (Zhang et al., 2006) or peptide pro-drugs (Ezra et al., 2000), have been explored without advancing the clinical use of these drugs. Formulation techniques using liposome encapsulation of the parent N-BP compound have also been explored as a means of improving oral bioavailability and cell-membrane permeability (Shmeeda et al., 2010; Caraglia et al., 2013). In addition, a number of structural modifications aiming to reduce the acidity of the bisphosphonate pharmacophore, increase the overall lipophilicity of a compound and/or decrease the affinity for bone, have been explored. Replacement of the Cα-hydroxyl with a hydrogen (e.g., **2a** vs. the corresponding deoxybisphosphonate **2b**) or Cα-halogen substituent (e.g., **2c**,**d**) has been found to decrease affinity for bone, as well as the potency in inhibiting hFPPS (Marma et al., 2007). Bisphosphonate inhibitors with various heterocyclic lipophilic side chains, including derivatives of zoledronic acid (e.g., **1b**,**c**) pyridinium deoxybisphosphonates (**7a–c**) (Zhang et al., 2009), aminopyridines (**8a–d**) (De Schutter et al., 2012; Lin et al., 2012), azaindoles (Ebetino et al., 2010a,b) and imidazopyridines (**9a**–**c**, respectively) (Ebetino et al., 2010c), and thienopyrimidines (e.g., **10**) (Leung et al., 2013a,b) have also been reported.

Although none of these analogs exhibit superior intrinsic potency in inhibiting hFPPS, as compared to the best current drugs (**1a**, **2a**), they exhibit improved cell-membrane permeability and lower bone affinity. For example, whereas the *in vitro* potency of analogs **8c**, **9b**, and **9c** is equivalent to that of the drug **2a** (all IC_50_ values ranging from 10 to 30 nM), these compounds exhibit lower affinity for bone. This observation is consistent with the hypothesis that the Cα-hydroxyl moiety, which is commonly referred to as the “bone hook” is absent in these analogs. Interestingly, the thienopyrimidine analog **10** was found to exhibit higher affinity for bone than **2a**, in spite of the absence of the Cα-hydroxyl (Leung et al., 2013b). Preliminary evidence of improved cell-membrane permeability by these more lipophilic analogs has been deduced mainly from cell-based assays measuring antiproliferation in various cancer cell lines. For example, analog **7c** was found to be ~100-fold more potent than **1a** in inhibiting the proliferation of breast cancer MCF-7 cells (Zhang et al., 2009), whereas analog **8b** was shown to be slightly more potent than **1a** in inhibiting the MM cell lines RPMI-8226 and KMS 28PE, in spite of a difference of nearly 10-fold in intrinsic potency (Lin et al., 2012). Inhibition of ERK phosphorylation, a signaling protein down-stream from Ras, was also observed with analog **8b** in KMS 28PE cells, providing evidence that this compound inhibits the intended biochemical pathway (Lin et al., 2012). At the present time, it is difficult to predict whether N-BPs with much higher lipophilicity and lower affinity for bone can provide sufficiently higher systemic exposure *in vivo* to significantly alter the clinical profile of such compounds in treating non-skeletal diseases.

**Figure:**
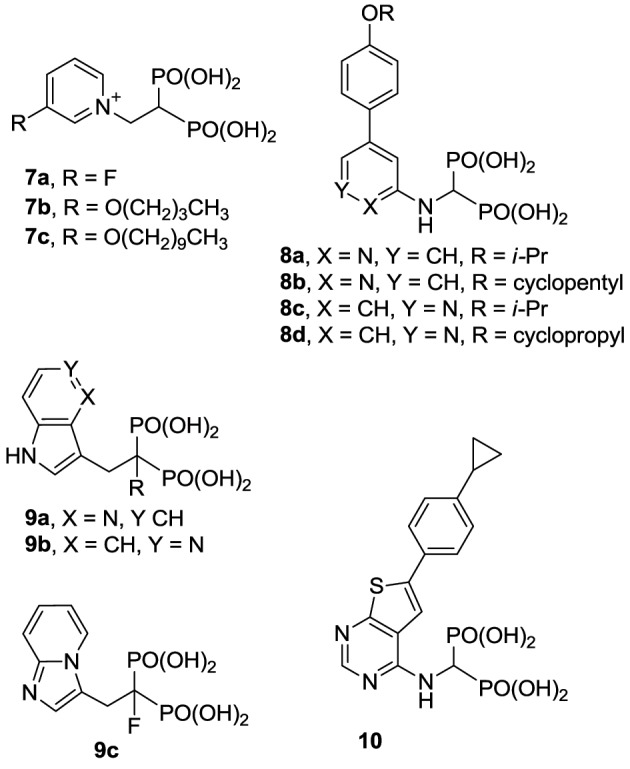


The structural diversity of N-BPs and their corresponding biological effects have also enriched our understanding of the molecular recognition requirements for potency and selectivity in inhibiting hFPPS vs. other functionally related enzymes. For example, the pyridinium analogs **7a** and **7b** are more selective in inhibiting hFPPS than hGGPPS, whereas derivatives with a longer alkyl substituent, such as **7c**, inhibit hFPPS, hGGPPS, and hDPPS with very similar potencies (reported IC_50_ values for **7c** in inhibiting hFPPS, hGGPPS, and hDPPS are 100, 280, and 585 nM, respectively) (Zhang et al., 2010). Crystallographic evidence with FPPS from *T. brucei* suggested that the first four carbons of the alkyloxy side chain can occupy the allylic sub-pocket, whereas, the longer analogs (e.g., **7c**) force the hydrocarbon chain to fold back, in order to avoid a steric clash with the protein surface of the allylic sub-pocket (Zhang et al., 2009). In contrast, the conformationally rigid analogs **8a,d**, and **10** bind in the allylic sub-pocket by forcing the expansion of the binding cavity toward the capping phenyl (F98/F99), while maintaining selectivity for hFPPS vs. hGGPPS; detailed characterization of the protein/ligand interactions are discussed in the following section.

### Allosteric inhibitors of hFPPS

Highly “druggable” enzymes (Cheng et al., 2007) are characterized by the presence of well-defined, rigid, hydrophobic pockets to which small molecules, with good “drug-like” properties (as defined by Lipinski's rule of five; Lipinski et al., 2001), can bind and block their catalytic activity. In contrast, the prenyl synthase enzymes hFPPS and hGGPPS are characterized by active sites with a high density of charged amino acid residues and significant conformational plasticity; such enzymes present a challenge for drug discovery and are often branded as “undruggable” (Verdine and Walensky, 2007; Crews, 2010). In fact, if it had not been for the serendipitous discovery (more than 40 years ago) that bisphosphonates have clinical value as inhibitors of bone loss, hFPPS could have easily been classified into this category of “undruggable” targets (Russell, 2011). However, increasing awareness of the pleiotropic effects that hFPPS inhibitors may have in human non-skeletal diseases, has stimulated efforts toward the identification of non-bisphosphonate inhibitors for this target. To date, non-bisphosphonate active site inhibitors of hFPPS have not been identified, and given the highly charged nature of the active site cavity, the design of such compounds is unlikely. Consequently, the focus has turned toward the identification of more “druggable” allosteric pockets of this enzyme.

Fragment-based screening by NMR and X-ray crystallography by Jahnke and co-workers at Novartis, led to the first discovery of compounds (e.g., **11**, **12**) that bind in an allosteric pocket of hFPPS near the IPP sub-pocket (Jahnke et al., 2010). Subsequent optimization of these hits provided non-bisphosphonate allosteric inhibitors with nanomolar potency (e.g., **13**, **14**). At the same time, Novartis reported quinolone (Amstutz et al., 2009) and salicylic acid (Cotesta et al., 2010) derivatives (e.g., inhibitor **15** and **16**, respectively) as inhibitors of hFPPS. Although the binding interactions of these compounds with the enzyme have not been disclosed, given their chemical structure, it is highly unlikely that they bind to the active site of hFPPS. Following these initial reports, *in silico* screening identified bisamidine-type inhibitors of hFPPS (e.g., **17**) (Lindert et al., 2013), although the binding mode of these compounds has also not been reported. In our own efforts, multistage biochemical and structural screening led to the discovery of thienopyrimidine analogs (e.g., **18**) that bind to the same allosteric pocket as compound **13** and exhibit equivalent *in vitro* potency (De Schutter et al., 2014).

In this account of examples from various structural classes of hFPPS inhibitors, we have purposely avoided making systematic comparisons of the *in vitro* potencies of compounds published by different research groups. Several different assays have been reported in the literature for evaluating hFPPS inhibition *in vitro*. The most commonly used method was originally developed by Reed and Rilling (1975) and has been adopted by many researchers with only minor modifications (Marma et al., 2007; Dunford et al., 2008; Lin et al., 2012); we refer to this assay as method 1 (M1). As expected, the IC_50_ value of any compound can vary considerably, depending on the assay method used for screening. For example, the reported IC_50_ values of zoledronic acid (**1a**) range from ~2–4 nM (Kavanagh et al., 2006a; Glickman and Schmid, 2007; Dunford et al., 2008) to 200 nM (Amstutz et al., 2009) and can be as high as 475 nM if the compound is tested without pre-incubation with the enzyme (Dunford et al., 2008). Experimental artifacts in a functional assay can further contribute to the observed potency of a compound and mislead SAR studies. Lipophilicity-dependent aggregation of moderately (or poorly) hydrophilic compounds is the most common factor responsible for false experimental data and can affect both *in vitro* and cell-based assays (Owen et al., 2012). The ability of bisphosphonates to form stable, high-order complexes in solution, particularly in the presence of metal ions, is a well-documented phenomenon, exploited in a number of biomedical applications (Kunnas-Hiltunen et al., 2009; Freire et al., 2010; Giger et al., 2013). Large lipophilic compounds, and particularly the non-bisphosphonate inhibitors, are more likely to suffer from solvent-induced hydrophobic interaction than the small polar N-BP drugs. A number of assay modifications have been reported that include lowering the concentration of Mg^2+^ ions and adding Triton X-100 in the assay buffer (Peukert et al., 2008; Lindert et al., 2013). Comparison of the intrinsic potencies of all the known hFPPS inhibitors is very challenging, even when compounds are tested under the same assay conditions. For example, biological profiling of a series of bisphosphonate and non-bisphosphonate inhibitors in the original M1 assay and a modified assay that we developed (method 2; M2) revealed minimal differences (<2-fold) in the IC_50_ values observed for the small bisphosphonates, such as **2a** and **8c**, but significant shifts of up to 20-fold with the lipophilic thienopyrimidine analogs (e.g., **10**) and the allosteric inhibitors **13** and **14** (Leung et al., 2013b).

**Figure:**
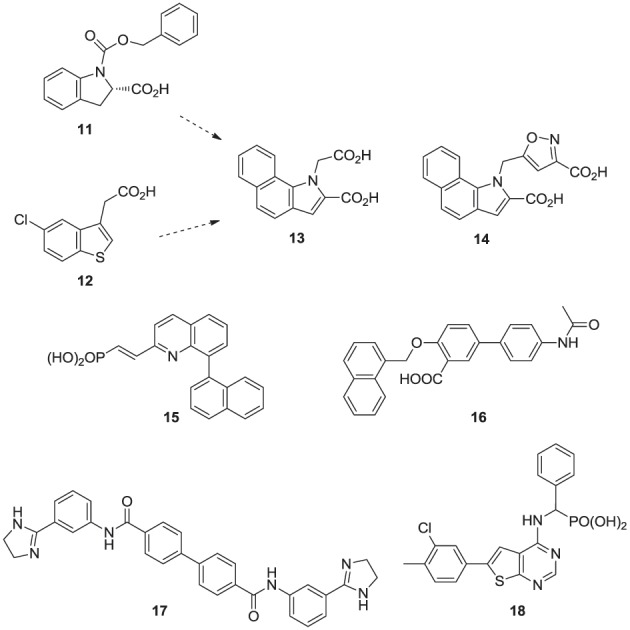


### Inhibitors of hGGPPS

Compared with the well-studied hFPPS, there is little information available on the structure of hGGPPS, and only a handful of selective inhibitors for this target have been reported. All of the known exploratory hGGPS inhibitors are bisphosphonates; however, none of the current bisphosphonate drugs targeting hFPPS, such as **1a** and **2a**, exhibit any significant potency in inhibiting hGGPPS. Bisphosphonates with long side chains, such as analogs **7c**, **19**, and **20**, have been shown to inhibit hGGPPS (Szabo et al., 2002; Zhang et al., 2009, 2010). Branched bisphosphonates, such as the digeranyl analog **21** (Shull et al., 2006; Barney et al., 2010) and the substituted biphenyl bisphosphonate **22**, have been described as selective hGGPPS inhibitors (Guo et al., 2007). The X-ray structure of the *S. cerevisiae* GGPPS/**22** complex showed that this inhibitor occupies the equivalent site as seen in the GGPP-bound hGGPPS structure (for a detailed discussion refer to the section below).

**Figure:**
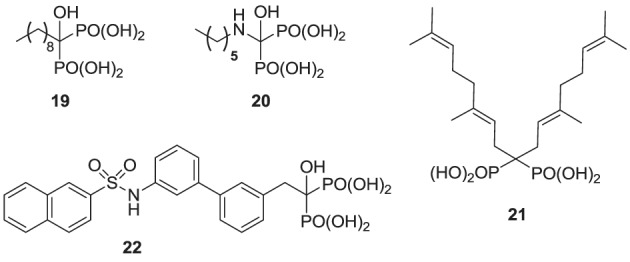


Given the substrate/product selectivity of hGGPPS (C_15_/C_20_ isoprenoids, respectively) it is not surprising that bisphosphonates with large side chains inhibit this enzyme. The reported potency of these compounds (IC_50_ values) is in the range of 0.3–3 μ M and none of the currently known hGGPPS inhibitors have been advanced to clinical development.

### Inhibitors of hSQS

Over the last two decades, various structural classes of squalene synthase inhibitors have been described. These include FPP substrate mimics, such as the bisphosphonate **23a**, its corresponding foscarnet derivative **23b** (Biller et al., 1991), the α-phosphonosulfonic acid **24** (Magnin et al., 1995, 1996; Lawrence et al., 1996), and aromatic bioisosteres, such as compounds **25**–**27** (Biller et al., 1993; Ciosek et al., 1993), Some of these analogs were shown to inhibit hSQS with low nanomolar IC_50_ potency. Furthermore, after intravenous and/or oral administration, some analogs lowered blood levels of cholesterol in rat and hamster, without affecting the levels of dolichol and coenzyme Q9 (Ciosek et al., 1993).

The second class of hSQS inhibitors includes mainly transition-state analogs that mimic the electrostatic and topological properties of the putative carbocation intermediates during the first and second steps in the catalytic conversion of FPP to squalene (Figure 2). The main structural requirements in the design of these compounds include the incorporation of a large lipophilic moiety stimulating the binding of the bulky and hydrophobic FPP hydrocarbon chain. In addition, the presence of a basic nitrogen, which is protonated at physiological pH, mimics the positive charge of the putative carbocation intermediate formed during the catalytic cycle; examples include the pyridine analog **28** (Prashad et al., 1993). Cyclopropyl amines, mimicking the structure of presqualene (Figure 2), have also been described (Poulter et al., 1989). Interestingly, the non-pyrophosphate derivative **29**, failed to inhibit yeast SQS in the absence of inorganic pyrophosphate (PPi), whereas in the presence of PPi it exhibited potent synergistic inhibition of the enzyme (Poulter et al., 1989). In contrast, the pyrophosphate derivative **30** was a potent inhibitor of SQS even in a pyrophosphate-free buffer, suggesting the absolute requirement of an ion pair during the rearrangement of presqualene pyrophosphate to squalene (Poulter et al., 1989).

**Figure:**
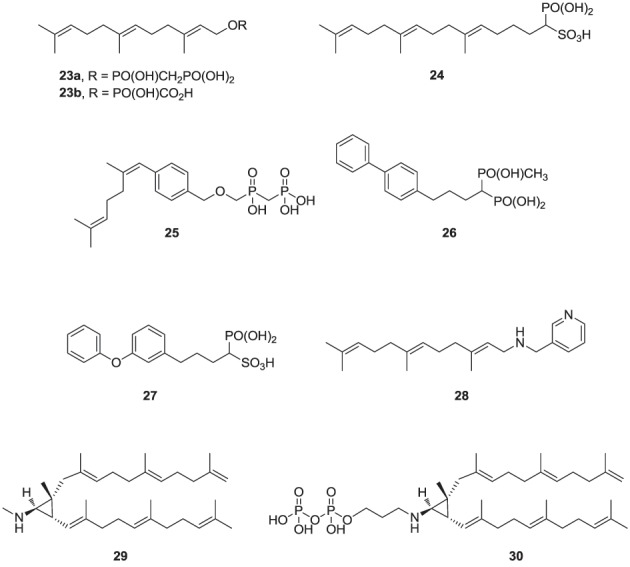


Numerous molecules that are simpler to synthesize and more drug-like, including the benzoxazole-based amines **31** and **32** (Ullrich et al., 1994; Morris et al., 1995), quinuclidine biphenyl or quinolone (e.g., **33** and **34**, respectively), and various other amine-based SQS inhibitors (e.g., **35**, **36**) have been explored. These molecules are also considered to act as mimics of the postulated cyclopropyl cation intermediate in the rearrangement reaction to squalene, a property that is attributed to their substituted amine being protonated under physiological conditions. However, the binding mode of these compounds remains unknown. Many of these analogs were reported to exhibit SQS inhibition activity in the low nanomolar or picomolar range (Brown et al., 1995). The requirement for the presence of PPi in the assay buffer (described above for analog **29**) was also observed with these inhibitors, making their *in vivo* potency somewhat unpredictable. Nonetheless structural optimization led to the identification of compounds with good biopharmaceutical properties, including reasonable oral bioavailability (Ishihara et al., 2004). Quinuclidine derivatives in particular, such as **33** and **34** (Brown et al., 1996), exhibited an equal or better pharmacological profile to statins in lowering cholesterol levels in hamsters, guinea pigs and Rhesus monkeys (Amin et al., 1997; Brown et al., 1997). However, none of these compounds were advanced to clinical trials; it is noteworthy that the quinuclidine structural motif is believed to be responsible for the toxicities associated with antimalarial drugs, such as quinine.

**Figure:**
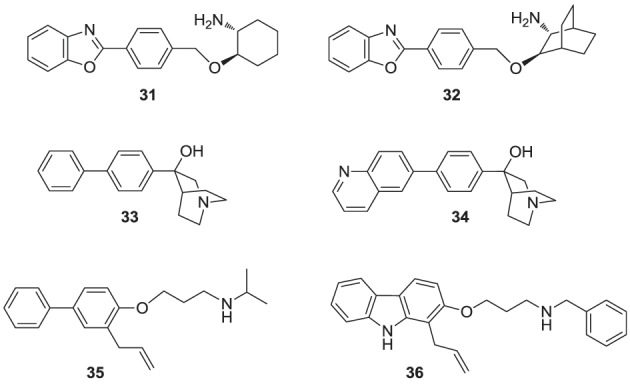


Natural products are a rich source of therapeutic agents and lead structures for drug discovery. Zaragozic acids A (**37**), B, and C are a family of fungal metabolites that have been shown to inhibit hSQS (Baxter et al., 1992; Bergstrom et al., 1993). However, and in spite of extensive SAR studies (Chan et al., 1996), the overall poor biopharmaceutical properties of these compounds prevented their development (Vaidya et al., 1998). Similarly, dicarboxylic acid amphiphilic molecules, bearing structural resemblance to zaragozic acids, have been explored. Examples include analogs **38** and **39**, which are potent inhibitors of hSQS *in vitro*, and can induce lowering of plasma cholesterol after oral administration in animal models (Iwasawa et al., 1995; Fung et al., 1997); further development of these compounds has not been reported.

**Figure:**
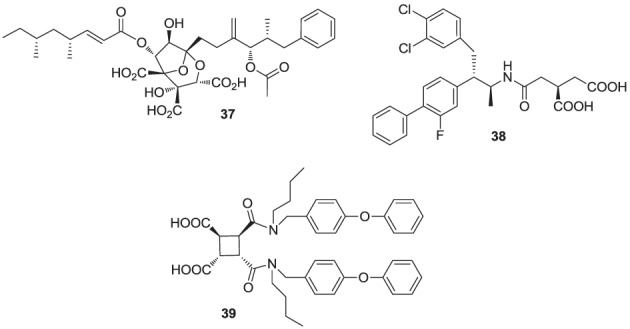


It has been proposed that statins are remarkably effective in preventing cardiovascular events due to their combined cholesterol lowering and antioxidant activity (Franzoni et al., 2003). Inhibitors of the hSQS that are also endowed with antioxidant and/or anti-inflammatory properties have been reported to exhibit significant antihyperlipidemic and antiatherosclerotic activity *in vivo* (Tavridou et al., 2007). The first generation of such compounds is represented by the morpholine derivative **40** (EP2302) that combines antioxidant properties and hSQS inhibition activity *in vitro*. It was reported that intraperitoneal administration of **40** leads to significant decrease in total cholesterol, low density lipoprotein, and triglycerides in the plasma of Triton WR-1339 induced hyperlipidemic rats (Chrysselis et al., 2000; Tavridou et al., 2008). Additional pharmacokinetic studies of the modified derivative **41** revealed a 6 to 7-fold higher accumulation of this compound in the liver (the main target tissue for hSQS activity) vs. the blood (Matralis et al., 2011).

Recent studies suggest that loss of endothelium-derived NO activity may contribute to the atherogenic process that includes monocyte adhesion to the endothelial surface, platelet aggregation, vascular smooth muscle cell proliferation, and vasoconstriction (Laufs et al., 1998). Morpholine derivatives that could inhibit hSQS in addition to releasing nitric oxide, such as compound **42**, were also explored (Chrysselis et al., 2002; Tavridou et al., 2007). Furthermore, SAR studies of this series of compounds include efforts to incorporate structural features of known antioxidant and/or anti-inflammatory, and even antidiabetic pharmacophores; examples include incorporation of the antioxidant phenothiazine core (e.g., analog **44**) and the side chain of the non-steroidal anti-inflammatory agent naproxen (e.g., analog **43**) (Kourounakis et al., 2010; Ladopoulou et al., 2013; Matralis and Kourounakis, 2014).

**Figure:**
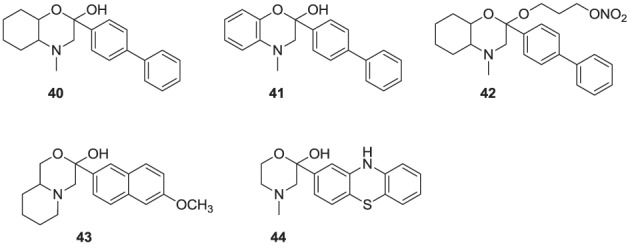


Finally, high throughput screening identified fused heterocyclic compounds, typified by the benzoxazepine analog **45**, as inhibitors of the SQS enzymes from rat and hepatocellular carcinoma HepG2 cells (Miki et al., 2002a). SAR optimization led to the discovery of analog **46a**, with low nanomolar potency in inhibiting both rat and HepG2 SQS (Miki et al., 2002b). Pharmacokinetic investigations revealed that the primary metabolite of **46a** is the CYP-450 product **46b**, which is rapidly eliminated in the urine, but is also active in inhibiting SQS. Structural modifications focusing on overcoming metabolic liabilities without loss of potency were successful in identifying the clinical candidate TAK-475 (**47b**). Although the binding mode and molecular interactions of TAK-475 with hSQS have not been reported, co-crystal structures of structurally related analogs have been reported and will be discussed in a later section.

TAK-475 and its main metabolite, the deacetylated compound **47a**, are both competitive inhibitors of hSQS with respect to farnesyl pyrophosphate and exhibit good tissue distribution to the liver (Miki et al., 2002c). TAK-475 was found to enhance the binding of ^125^I-LDL to the LDL receptor and increase LDL receptor expression in a manner similar to that observed with statins. A decrease in total cholesterol and triglyceride plasma levels was also observed in marmosets when TAK-475 was administered at doses of 30 and 100 mg/Kg without affecting HDL levels (Nishimoto et al., 2003a). TAK-475 also delayed the progression of coronary atherosclerosis in a rabbit model; interestingly, changes of macrophage/lipid rich atheromatic plaques (that are prone to rupture) into stable fibromuscular lesions were observed and attributed to the lowering of oxidized lipoprotein content in the plaques and an increase in CoQ10 levels (Shiomi et al., 2008). *In vitro* testing in human rhabdomyosarcoma cells and human skeletal muscle cells suggested that TAK-475 did not induce myotoxicity [the most common side effect of statins, associated with depletion of mevalonate derived isoprenoids (Nishimoto et al., 2003b)], and surprisingly when co-administered with statins to guinea pigs, TAK-475 seemed to decrease statin-induced myotoxicity (Nishimoto et al., 2007). In spite of all the favorable properties of TAK-475 (**47b**), lack of superiority in clinical benefits as compared to current standard of care (i.e., treatment with statins), in addition to liver toxicity at the dose required to achieve efficacy, led to termination of its clinical development (Stein et al., 2011). Nonetheless, SQS is still considered a viable target for drug discovery in the treatment of atherosclerosis (Liao, 2011) and new derivatives of TAK-475, such as the tricyclic pyrrole derivative **49**, are still under investigation (Ichikawa et al., 2013).

**Figure:**
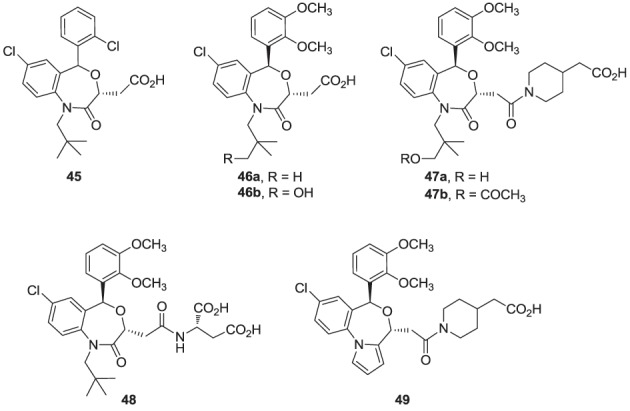


## Structures of prenyl synthase enzymes—implications for drug discovery

Structure-based rational drug design provides a creative, efficient alternative to high-throughput screening approaches commonly used in industry for the identification of hit or lead compounds (Lounnas et al., 2013). Since this approach relies on detail knowledge of the molecular recognition elements between ligands and targets, three dimensional atomic structures of potential therapeutic targets, particularly in complex with ligands/inhibitors, are highly valuable. Owing to on-going technological advancements in the methods used in macromolecular structure determination, increasing numbers of pharmaceutical targets are becoming amenable to structure-based approaches (Thiel, 2004). The prenyl synthase enzymes, particularly hFPPS, undergo significant conformational changes during catalysis and are involved in “induced fit” binding interactions with their inhibitors; typically, such properties make structure-based drug design more challenging. Nonetheless, detail knowledge of the molecular interactions between inhibitors of these enzymes and their binding pockets is indispensable in guiding medicinal chemistry efforts.

### Human FPPS

Numerous co-crystal structures of hFPPS are currently available, in complex with various ligands, including the best current N-BP clinical drugs. These structures have provided tremendous insights into the catalytic cycle, protein-ligand binding interactions, and future drug design strategies for this target. Here, we aim to summarize the great wealth of structural information presently available on hFPPS in complex with various inhibitors.

### Overall structure

**Figure 3:**
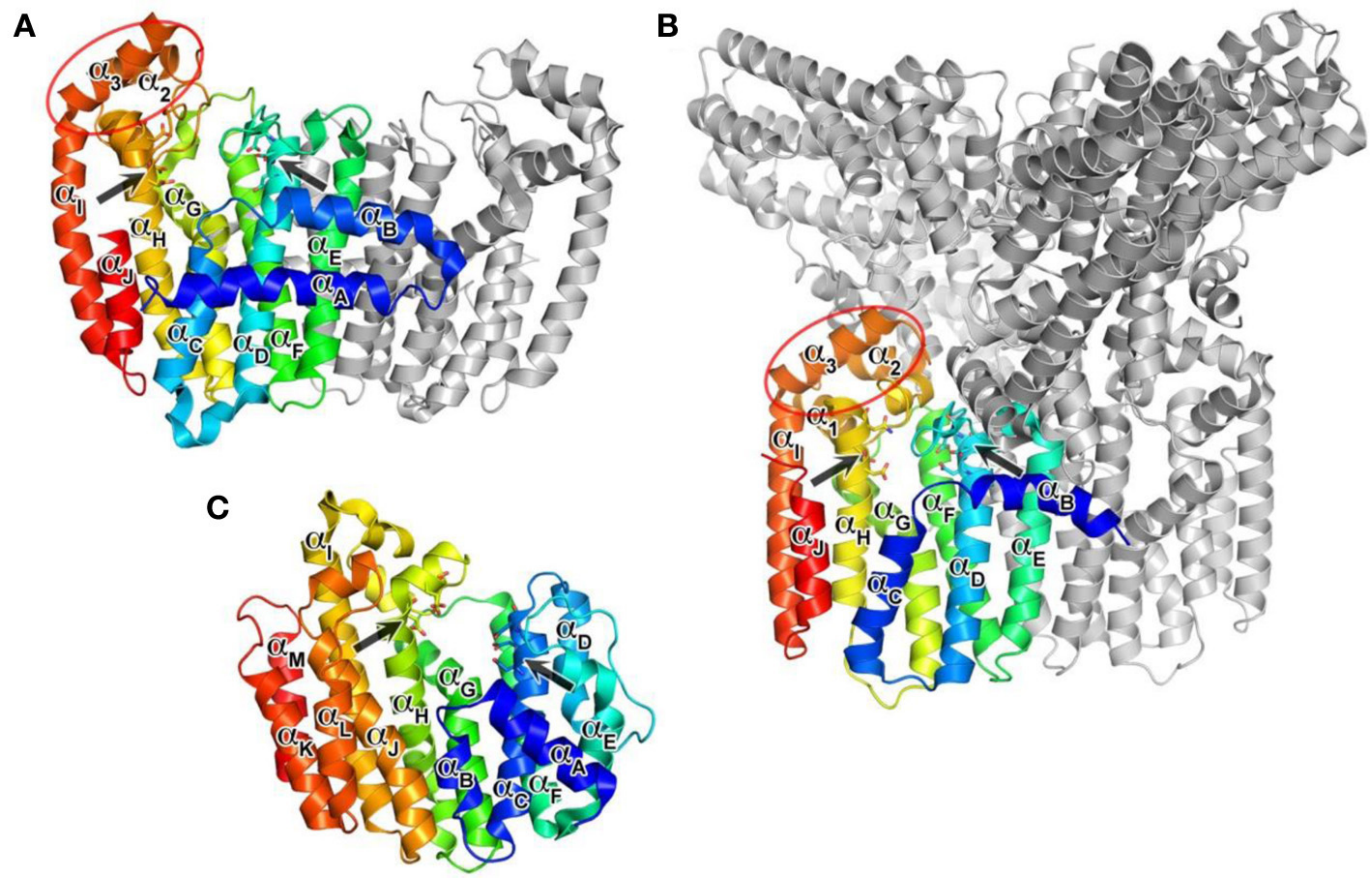
**Overall structures of human prenyl synthase enzymes. (A)** Farnesyl pyrophosphate synthase (PDB ID: 2F7M); **(B)** geranylgeranyl pyrophosphate synthase (PDB ID: 2Q80), and **(C)** squalene synthase (PDB ID: 3WEG). The enzymes are shown in a blue-to-red rainbow color scheme. For hFPPS and hGGPPS, one monomeric subunit of the enzyme oligomer is represented in color. The conserved aspartic-acid rich motifs are shown in a stick representation and marked by arrows. The small domains composed of α_1_ − α_3_ in hFPPS and hGGPPS are indicated by elliptical circles; in this view, the α_1_ in hFPPS is hidden behind the top portion of α_H_.

The structure of hFPPS was first determined in 2006, simultaneously by two independent groups (Kavanagh et al., 2006a; Rondeau et al., 2006). The enzyme exists as a homodimer of a 41 kDa subunit and has an all α-helical structure, a feature also shared by hGGPPS and hSQS (Figure 3). The hFPPS monomer is comprised of ten core helices (α_A_ − α_J_), constituting its main body around a central catalytic cavity, and three short helices (α_1_ − α_3_) inserted between α_H_ and α_I_ that form a small peripheral domain (Figure 3A). This domain, which is absent in prokaryotic FPPS (Hosfield et al., 2004), may function as a binding interface for interaction with other proteins. The two aspartate-rich (DDXXD) motifs conserved across all species and also in GGPPS are located at the end of α_D_ and α_H_, and face the central cavity from opposite sides (Figure 3A). These motifs are followed immediately by extended loop regions (Ser108-Asp127 and Leu248-Ser268), which shape a large portion of the outer perimeter of the active site cavity. Overall, the ligand-free form of the hFPPS monomer can be described as consisting of two similarly sized lobes separated by a large deep cleft containing the active site. Enzyme dimerization is mediated by the N-terminal lobe, with the dimer interface formed mainly by α_E_ and α_F_.

### Allylic substrate binding site

**Figure 4:**
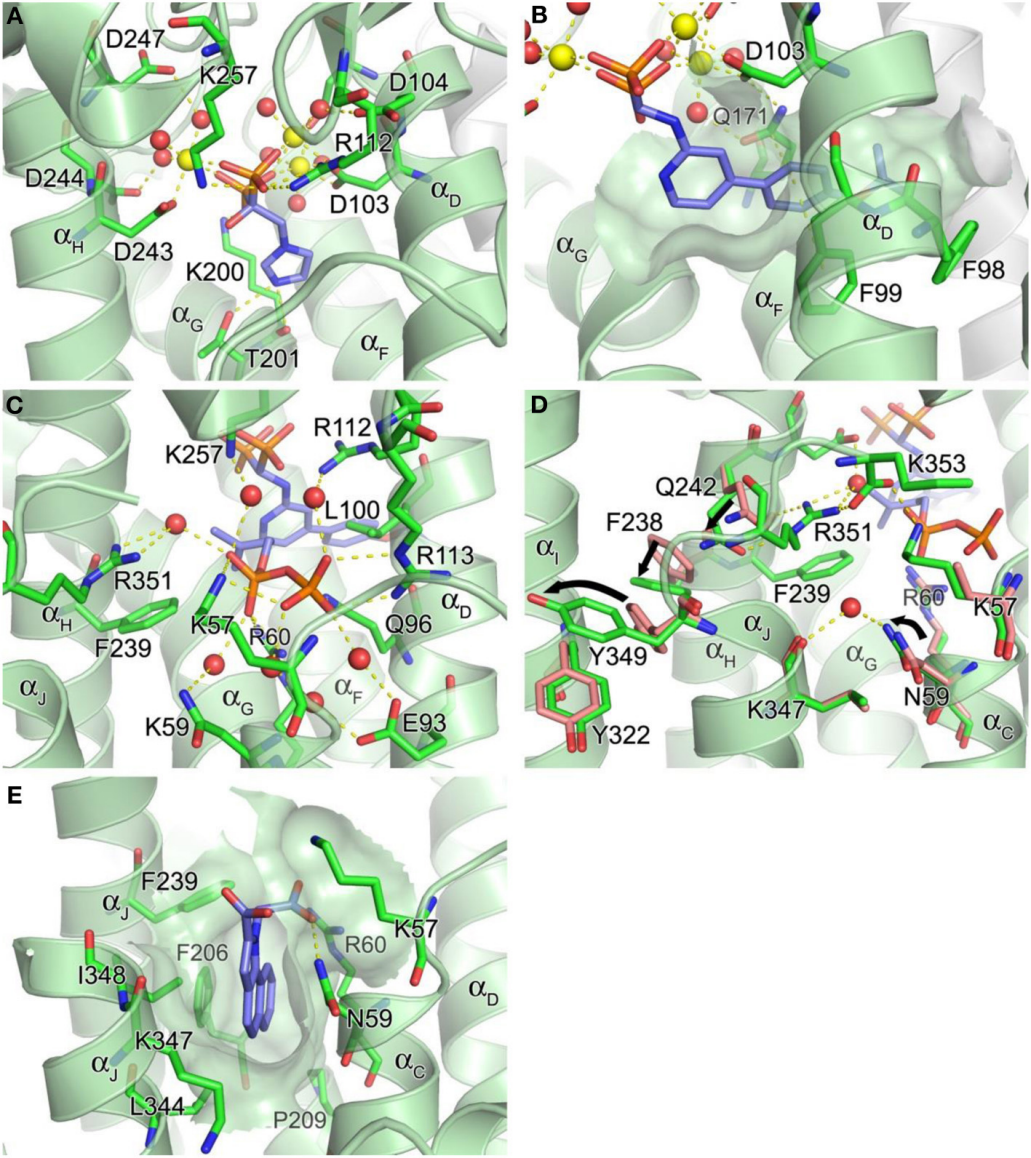
**Structure of human farnesyl pyrophosphate synthase. (A)** Binding of zoledronate (**1a**) at the allylic site (PDB ID: 2ZW5). Mg^2+^ ions and water oxygen atoms are shown as yellow and red spheres, respectively. Yellow dashes indicate molecular interactions. **(B)** Binding of compound **8a** (PDB ID: 4DEM). The hydrophobic channel accommodating the side chain of the inhibitor is rendered in a semi-transparent surface representation. **(C)** Binding of IPP at the homoallylic site (PDB ID: 4H5E). **(D)** Conformational changes responsible for structuring the C-terminal tail. **(E)** Binding of compound **13** at the allosteric site (PDB ID: 3N6K).

The DDXXD motifs in hFPPS are essential for the binding of the allylic substrates DMAPP and GPP. The substrate ligation occurs primarily through three Mg^2+^ ions and metal-coordinated water molecules that mediate the binding interactions between the aspartate residues and the pyrophosphate of the isoprenoids. The conserved residues Arg112, Lys200, and Lys257 also contribute to the substrate binding by forming direct salt bridges with the pyrophosphate. The bisphosphonate inhibitors of hFPPS act competitively with respect to the allylic substrates and bind to the enzyme in an analogous manner, with their bisphosphonate moiety mimicking the pyrophosphate of the substrates (Figure 4A). Substrate/inhibitor binding brings the DDXXD motifs closer together, resulting in a major conformational change involving a rigid body motion of the entire C-terminal lobe. The structural transition drastically reduces the volume of the centrally located cavity and shuts its opening to the allylic substrate binding site, forming a discrete pocket around the trinuclear Mg^2+^ cluster. It is currently unknown whether Mg^2+^ ions and the ligands (i.e., the allylic substrates or bisphosphonate inhibitors) bind to hFPPS sequentially or as a preformed cluster.

In addition to the 3Mg^2+^/pyrophosphate (or bisphosphonate) binding area, the allylic substrate sub-pocket consists of a deep hydrophobic channel that accommodates the isoprenyl hydrocarbon tail of the substrate or the side chain of a bisphosphonate inhibitor (Figure 4B). The channel extends to the enzyme's dimerization interface, where the residues Ile143, Asn144, and Asn147 of the adjacent monomer form a dead-end wall. Asp103, the first aspartate residue of the N-terminal DDXXD motif, is found at the opening of this channel and likely functions as its gatekeeper. Without the Mg^2+^ ions bound to the conserved motif the side chain of Asp103 points into the opening, rendering it too narrow to accommodate an isoprenyl chain or a structurally similar mimic (e.g., PDB IDs: 2F7M and 2RAH). Other residues of importance for forming this channel include Phe98, Phe99, and Gln171 (Figure 4B). Besides providing a hydrophobic surface for the tail of the allylic substrate, the phenylalanine residues play a key role in determining the length of the final product's hydrocarbon chain (i.e., C_15_ for FPP) (Tarshis et al., 1996). Gln171 also facilitates binding of the allylic tail via π-stacking interaction involving its planar side chain, but this residue further contributes to substrate binding by providing a polar contact to the pyrophosphate-Mg^2+^-water cluster. In our work, we have shown that these residues participate in similar interactions with bisphosphonate inhibitors that have extended and rigid lipophilic side chains, such as the aminopyridine inhibitors **8a** and **8b** (Figure 4B) (De Schutter et al., 2012; Lin et al., 2012), and the thienopyrimidine-based inhibitor **10** (Leung et al., 2013b).

The hFPPS catalysis is thought to proceed as a three-step ionization-condensation-elimination reaction in which the nucleophilic tail double bond of IPP attacks the C1 atom of the allylic substrate (i.e., DMAPP or GPP) (Cornforth et al., 1966; Poulter et al., 1978). The initial ionization of the allylic substrate is triggered by the bound Mg^2+^ ions and generates a pyrophosphate as well as an allylic carbocation with its charge distributed over the C1, C2, and C3 atoms. The carbocation intermediate is stabilized through electrostatic interactions with the liberated pyrophosphate and polar interactions with the main chain carbonyl oxygen of Lys200 and the side chain oxygens of Thr201 and Gln240 (Hosfield et al., 2004). As discussed earlier, the oxygen atoms of Lys200 and Thr201 form a bifurcated H-bond with the protonated heterocyclic nitrogen of the most potent current N-BP drugs, minodronate (**3**, PDB IDs: 2VF6; 3B7L), zoledronate (**1a**; Figure 4A; PDB IDs: 1ZW5, 2F8C, 2F8Z, 2F9K), and risedronate (**2a**, PDB ID: 1YQ7; 1YV5), which is presumed to be protonated in the enzyme bound state. N-BPs with alkyl amine side chains, such as pamidronate (**4**, PDB ID: 2F89) and alendronate (**5**, PDB ID: 2F92), are significantly less potent (>50-fold loss in potency as compared to **1a**, **2a**, or **3**). In the past, this loss in potency was attributed (*at least in part*) to the preference for having a nitrogen atom four bonds away from the Cα for best alignment and H-bond distance from the oxygens of Lys200 and Thr201 in the bound state. However, the potency of these compounds is affected by multiple factors, including the basicity of the nitrogen-containing side chains and the energy required for desolvation of the side chain before binding to the enzyme; the latter effect may account in part of the potency difference between the primary amine analog pamidronate (**4**) and the much more lipophilic tertiary amine derivative ibandronate (**6**; the nitrogen atom is three σ-bonds away from the Cα in this compound). Additional interactions of the longer side chain of **6** with the lipophilic region consisting of the capping phenyls, is accompanied with minor conformational changes to the allylic sub-pocket (PDB ID: 2F94); ibandronate is only ~5-fold less potent than the most potent N-BP drugs (Dunford et al., 2008).

### IPP binding sub-pocket (homoallylic substrate binding site)

The IPP binding sub-pocket is only fully shaped upon occupancy of the allylic sub-pocket which results in the previously described conformational change that partially closes the active site cavity. This sub-pocket is composed mainly of basic residues, including the highly conserved residues, Lys57, Arg60, and Arg113, which form direct salt bridges with the pyrophosphate of the bound IPP. Three other residues, Arg112, Lys257, and Arg351, participate in indirect interactions through water molecules (Figure 4C). Additional interactions to the IPP pyrophosphate at this site include a direct H-bond with Gln96 and water-mediated H-bonds with Asn59 and Glu93 (Figure 4C). Conserved residues Leu100 and Phe239, on the other hand, provide hydrophobic contacts to the homoallylic tail of IPP (Figure 4C). These interactions position the tail double bond of IPP close to the C1 atom of the allylic substrate, thus facilitating the nucleophilic attack during catalysis. The IPP tail likewise makes tight van der Waals contacts with the side chain of a bisphosphonate inhibitor. This interaction further stabilizes the bound inhibitor thus contributing to potency.

### hFPPS C-terminal tail

The initial occupancy of the allylic sub-pocket induces a protein conformational change from the “open” to a “partially-closed” state, which fully defines the shape of the IPP binding site. The subsequent binding of IPP leads to folding and rigidification of C-terminal tail residues (^350^KRRK^353^) over the IPP binding site (Figure 4D), completely closing the active site cavity and sequestering both substrates from bulk water (Song and Poulter, 1994). While hFPPS cycles through its three conformational states during catalysis, with chemically stable bisphosphonate inhibitors the ternary enzyme complex stalls in the fully closed state. This nearly irreversible inhibition of the enzyme is partly responsible for the clinical efficacy of the bisphosphonate compounds (Räikkönen et al., 2011).

The C-terminal tail closure of hFPPS is most notably characterized by rigidification of the Arg351 side chain, which anchors itself to the α_H_, in addition to forming a salt bridge with the terminal residue Lys353 (Figure 4D). The anchoring of Arg351 requires a cascade of preceding conformational changes in the residues Gln242, Phe238, and Tyr349. Tyr349 here likely functions as a safety switch preventing futile tail closure in the absence of bound IPP. When the side chain of Tyr349 is locked in the “off” position, the downstream conformational changes are inhibited by steric hindrance (Figure 4D). The IPP-induced tail closure is intriguing because the ligand when bound does not make any direct contact with either the tail residues or the switch residue Tyr349. A putative mechanism as to how the subtle conformational change triggered by IPP binding at the IPP sub-pocket translates into such a drastic movement of Tyr349 (i.e., the side chain rotation of ~80°, Figure 4D) has been proposed recently (Park et al., 2012): the pyrophosphate of IPP attracts and structures the side chain of Lys57, while pushing back Arg60; the displacements of these residues result in a ~15° rotation of Asn59, which in turn interacts with Lys347 through a recruited water molecule (Figure 4D). The ordered side chain of Lys57, on the other hand, attracts the enzyme's C-terminal tail by forming a salt bridge with the terminal carboxyl group (Figure 4D). The force applied simultaneously to the residues upstream and downstream of Tyr349 by these interactions may create a torque strong enough to rotate its side chain out of its “off” conformation.

Interestingly, inorganic pyrophosphate (PPi) can also bind to the homoallylic site of hFPPS and elicit the tail-closing conformational change in the presence of a bound bisphosphonate (Park et al., 2012). An implication of this observation is that PPi, which is a prevalent cellular metabolite, may have a relevant role in stabilizing an enzyme-inhibitor complex *in vivo*. On the contrary, inorganic phosphate (Pi) can bind to the IPP binding site (up to two molecules simultaneously), but does not appear to induce this conformational change (Park et al., 2012, 2014). Other anions observed to bind to the IPP sub-pocket of hFPPS include sulfate (PDB IDs: 2RAH; 3RYE; 3S4J; 4JVJ); similarly, bound acetate has been observed with FPPS from *T. brucei* (PDB IDs: 2OGD, 3DYG; Zhang et al., 2009). It is currently unknown whether the binding of Pi or other negatively charged cellular metabolites is physiologically relevant. However, it is conceivable that any non-substrate compound binding at the IPP sub-pocket would also inhibit the enzyme by competing with IPP. Indeed, *in vitro* inhibition of porcine FPPS by Pi (*albeit* at a high concentration) has been reported (Holloway and Popjak, 1967). In this light, it is noteworthy that cross-binding of isoprenoid substrates, such as binding of IPP to the allylic site of hFPPS (Kavanagh et al., 2006a) and of DMAPP to the homoallylic site of *T. cruzi* FPPS (Gabelli et al., 2006), have also been reported. However, no bisphosphonate or other small molecular compound that targets specifically the IPP binding site has been identified.

### Allosteric pocket

Recently, non-bisphosphonate inhibitors of hFPPS have been identified by a fragment-based, structure-guided approach combining NMR spectroscopy and X-ray crystallography (Jahnke et al., 2010). These inhibitors bind in a newly identified pocket adjacent to the IPP sub-pocket and near the C-terminal end of the enzyme (Figure 4E). This pocket is lined with α_C_, α_G_, α_H_, and α_J_, and is largely hydrophobic with contributions from the entire side chains of Phe206, Pro209, Phe239, Lue344, and Ile348, as well as the alkyl portions of the Asn59, Arg60, and Lys347 side chains. The initial fragment hits and the optimized compounds all contain a bicyclic or tricyclic heteroaromatic system with at least one carboxylic acid group. Most of these compounds bind through hydrophobic and/or π-stacking interactions between their heteroaromatic groups and the protein residues within this pocket. In addition, one of the carboxylic acid moieties of these compounds forms electrostatic interactions with the side chains of Arg60 and Lys57, as well as an H-bond with the side chain of Asn59 (Figure 4E). The mechanism of action of the allosteric inhibitors is thought to involve blocking the C-terminal tail from closing, inhibition of IPP binding due to electrostatic repulsion (between the carboxylic acid moiety and the pyrophosphate moiety) and disruption of the overall dynamics of the enzyme's catalysis cycle.

The discovery of the hFPPS allosteric pocket was unforeseen, since in the absence of a bound ligand, the pocket is small and only widens upon ligand binding via an induced-fit mechanism. It is presently unknown whether this pocket has a natural physiological role and whether it is truly highly druggable; proof of the latter will have to await the identification of very potent inhibitors of hFPPS that bind to this pocket and inhibit hFPPS *in vivo*.

## Human GGPPS—overall structure

In contrast to hFPPS (presently, 36 released and 20 unreleased PDB entries), there has been only one structure of hGGPPS reported so far (Kavanagh et al., 2006b). Like its paralog hFPPS, hGGPPS adopts an all α-helical fold (Figure 3B), and the monomer associates into a homodimer. However, three dimers of this enzyme join together to form a homohexamer complex, which has been described as a three-blade propeller (Figure 3B). Biochemical investigations have even described the formation of octameric complexes in solution (Miyagi et al., 2007). Despite low sequence identity (17%), the tertiary structure of hGGPPS is remarkably similar to that of hFPPS (Figure 3A). The two aspartate-rich (^64^DDIED^68^ and ^188^DDYAN^192^) motifs are found at locations equivalent to those in hFPPS, also facing the central catalytic cavity from opposite sides (Figure 3B); however, the C-terminal motif is replaced by DDXXN. Additionally, hGGPPS is a smaller protein than hFPPS, it has an extended loop region following this C-terminal motif that contains a short α-helical element (Figure 3B) and lacks the N-terminal helix α_A_ found in hFPPS (Figure 3B). Other secondary structure elements of hGGPPS are analogous to those described for hFPPS earlier, namely α_B_ − α_J_ and α_1_ − α_3_. Notably, the small domain composed of α_1_ − α_3_ in hGGPPS is involved in forming the inter-dimer contacts with α_B_ and the first extended loop region of the adjacent subunit (Figure 3B). The dimerization interface is formed mainly by α_E_ and α_F_ from each monomer in a two-fold symmetry-related fashion as also observed in hFPPS.

### Inter-dimer interface

**Figure 5:**
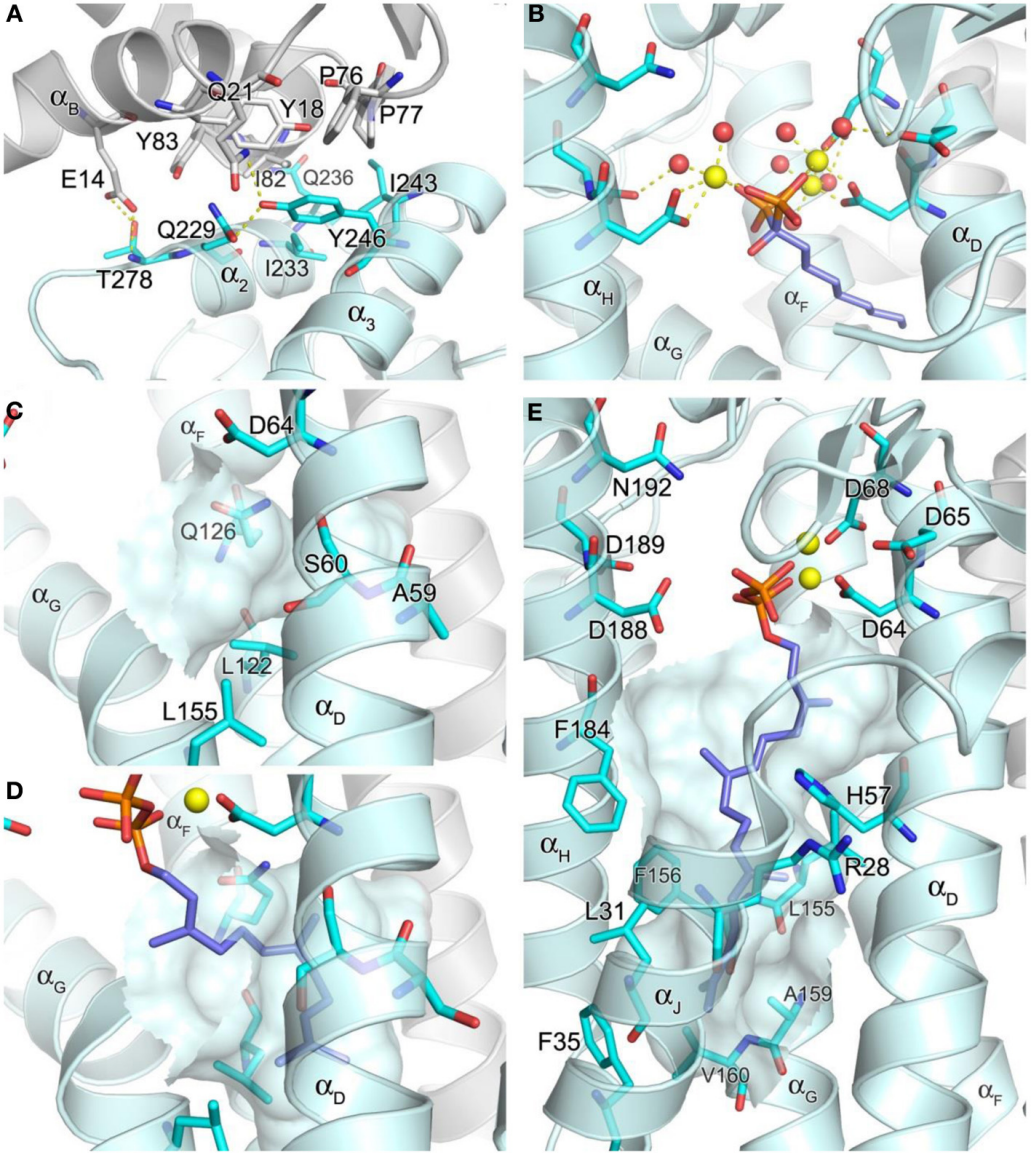
**Structure of geranylgeranyl pyrophosphate synthase. (A)** The inter-dimer interface in hGGPPS (PDB ID: 2Q80). **(B)** Binding of a bisphosphonate with a long alkyl side chain to yeast GGPPS (PDB ID: 2Z4X); the trinuclear metal cluster is similar to that in Figure 4A. **(C)** The product chain elongation channel in hGGPPS. **(D)** Binding of the substrate FPP in yeast GGPPS (PDB ID: 2E90); conformational changes of the key residues (as compared to the same residues in **C**) are evident; also Ala59 in hGGPPS is replaced with a serine in yeast GGPPS. **(E)** Binding of GGPP at the product inhibitory site in hGGPPS.

The core of the inter-dimer interface is composed of a hydrophobic patch formed by the small domain residues Ile233, Ile243, and Tyr246, and the N-terminal residues Tyr18, Phe76, Pro77, Ile82, and Tyr83 of the contacting subunit (Figure 5A). This contact is further stabilized by the H-bond interactions between Thr228 and Glu14, Gln236 and Ile82, and Tyr246 and Gln21 (Figure 5A). There are six such contacts in the entire hexamer, with each monomer contributing both the small domain and the N-terminal interactions. The residues involved in the inter-dimer contacts are conserved in mammalian and *Drosophila* GGPPS but not in plant, fungal, archaeal, or bacterial orthologs (Kavanagh et al., 2006a). This observation suggests that the hexameric organization of GGPPS quaternary structure is likely characteristic only of the mammalian and insect enzymes. All the other orthologs whose structures have been determined, including fungal and bacterial forms, display a dimeric organization. The relative abundance of structural information available on GGPPS from other organisms (>40 PDB entries released), as well as the low resolution (2.70 Å) of the reported human structure, suggests that the human enzyme is difficult to crystallize perhaps due to its hexameric nature. A large part of the discussion below therefore relies on known structures of other GGPPS orthologs, particularly those of the yeast enzyme (Guo et al., 2007; Zhang et al., 2009), as well as hFPPS.

### Catalytic cavity

Both the allylic (FPP) and homoallylic (IPP) substrate binding sites of hGGPPS are highly comparable to those previously discussed for hFPPS. The allylic substrate site is capped by the DDXX(D/N) motifs and the proceeding loop regions. FPP binding is thought to occur in a similar arrangement as observed for the binding of DMAPP/GPP in hFPPS, where three Mg^2+^ ions mediate the interaction between the side chains of the acidic residues and the pyrophosphate moiety. The basic residues that form direct contacts with the GPP/DMAPP substrate in hFPPS are also conserved for the binding of FPP in hGGPPS (i.e., Arg73, Lys151, and Lys202). The exact details of the metal mediated charge interaction, however, are unclear from the available structural model (PDB ID: 2Q80); in this case, only two Mg^2+^ ions are observed bound to the N-terminal DDXXD motif, and there are no water molecules coordinated with them. In addition, the Mg^2+^ ion interacting with the second DDXXD motif observed in hFPPS is missing entirely in this hGGPPS structure. While these discrepancies are probably due to the resolution limitation of the current hGGPPS structure, the higher resolution structures of the yeast enzyme also show varying numbers of chelated Mg^2+^ ions (i.e., 0–3), as well as diverse water coordination patterns. The reason for these inconsistencies may also lie in the fact that all of the substrate and inhibitor ligands bound to the yeast enzyme were introduced by soaking rather than co-crystallization. Alternatively, buffer pH effects resulting in protonation of the conserved aspartic acid residues and thus affecting the metal binding have also been suggested (Guo et al., 2007). Nonetheless, two structures of yeast GGPPS (PDB IDs: 2Z4X and 2Z52) show three Mg^2+^ ions and octahedrally coordinated water molecules mediating bisphosphonate binding in a manner analogous to that observed for hFPPS (Figure 5B).

The location and nature of the hydrophobic channel accommodating the allylic substrate's isoprenyl C_15_ tail in hGGPPS are also similar to those for hFPPS. However, in hGGPPS Ala59 and Ser60 replace the capping phenyls of Phe98/99 in hFPPS (that limit the growing chain length to C-15), thus allowing space for the C_20_ isoprenoid product of this enzyme (Figure 5C vs. Figure 4B). In addition, three nearby leucine residues (Leu56, Leu122, and Leu155), undergo rotational changes upon substrate binding and form the deepest corner of this hydrophobic channel to accommodate the end portion of the substrate's aliphatic tail (by analogy with yeast GGPPS; compare Figure 5C and Figure 5D).

The IPP substrate binding site in hGGPPS (like in hFPPS) is lined with basic residues that interact directly with the pyrophosphate of IPP (i.e., Arg28, His57, Arg73, and Arg74). Lue61 and Phe184 provide hydrophobic contacts to the IPP hydrocarbon tail and direct it toward the C-1 of the allyllic substrate. By analogy with hFPPS, hGGPPS catalysis is thought to proceed via the same ionization-condensation-elimination mechanism described previously. In support of this hypothesis, the three residues proposed to stabilize the allylic carbocation intermediate in hFPPS are all conserved in hGGPPS (i.e., Lys151, Thr152, and Gln185).

### Inhibitory conformation/pocket

It is notable that the only human GGPPS structure available shows a molecule of GGPP in complex with each monomer. Evidently, during heterologous bacterial expression of the human recombinant GGPPS enzyme, bacterial GGPP bound was retained though the enzyme purification and crystallization process (Kavanagh et al., 2006b). In this structure, the GGPP molecule does not represent the product state, as its pyrophosphate moiety is bound to the DDXX(D/N) motifs in the allylic site as opposed to the basic residues in the homoallylic site (IPP sub-pocket). Furthermore, its hydrocarbon tail extends down into a large, deep pocket below the active site (Figure 5E), and not into the hydrophobic channel believed to accommodate the growing isoprenyl tail of the product. This novel pocket has a hydrophobic surface, lined with the aliphatic and aromatic side chains of the residues Arg28, Leu31, Phe35, His57, Leu155, Phe156, Ala159, Val160, and Phe184. The enzyme-ligand complex here is thought to demonstrate a feed-back inhibitory state; this observation is consistent with GGPP and 3-azaGGPP acting as competitive inhibitor of hGGPPS with respect to the substrate FPP (Kavanagh et al., 2006a).

As a consequence of hGGPPS having two large hydrophobic sub-pockets in its active site cavity, the enzyme may allow binding of larger lipophilic bisphosphonates (e.g., compounds **21** and **22**) in multiple binding orientations. In the absence of co-crystal structures of the human GGPPS with inhibitors, co-crystal structures of the yeast GGPPS have shown diverse binding patterns for various compounds (Guo et al., 2007; Cao et al., 2008; Zhang et al., 2009). Some analogs bind in the allylic substrate site (similarly to the binding of small N-BPs in hFPPS), with their bisphosphonate moiety ligated to the DDXX(D/N) motifs and their side chains extended into the tail elongation site (e.g., PDB entries 2Z4X and 2Z52), whereas others bind like GGPP (in its inhibitory conformation) with their lipophilic side chains occupying the product inhibitor pocket (e.g., PDB entries 2Z7H and 2ZEV). In addition, compounds that bind simultaneously at two separate sites have been identified. With these bisphosphonates, one molecule binds at the allylic substrate site, and the other at the IPP binding site but with its bulky side chain in the inhibitory pocket (PDB IDs: 2E93 and 2Z4Y). Furthermore, bisphosphonate compounds that are branched at the Cα with long aliphatic groups bind to the allylic substrate site with one side chain in the tail elongation channel and the other in the inhibitory pocket (e.g., PDB: 2Z4W and 2Z4Z). In summary, the current structural information of GGPPS/ligand complexes suggest that there are many challenges to overcome in targeting the human enzyme and in understanding the molecular recognition elements required for the design of selective and potent inhibitors.

## Human squalene synthase (SQS)

### Overall structure

Human SQS (hSQS) is a 417 amino acid, 47-kDa enzyme localized at the endoplasmic reticulum, where it is anchored to the membrane with both of its terminal ends, while its larger catalytic domain is facing the cytosol. The crystal structure of the soluble, functional, doubly truncated form of hSQS (residues 31–370) was first determined by Pandit et al. (2000), and since then a number of additional structures with bound inhibitors have become available (Ichikawa et al., 2011, 2012; Liu et al., 2012). The protein is entirely α-helical (α_A_ − α_M_), and despite the lack of overall sequence homology, its core helices surrounding the central catalytic cavity (α_C_, α_F_ − α_H_) are structurally analogous to those of hFPPS and hGGPPS (α_D_, α_F_ − α_H_) (Figure 3C). The enzyme contains two conserved signature (DXXED) motifs, which are found on opposite walls of the catalytic cavity with the aspartate and glutamate side chains pointing into the cavity (Figure 3C). Of note, structural superposition shows that the DXXED motifs of hSQS occupy the same spatial locations as the DDXX(D/N) motifs of hFPPS and hGGPPS. The same chain fold and similar active site topology, as well as the conservation of functionally important residues, strongly suggest a distant evolutionary relationship between hSQS and hFPPS/hGGPPS.

### Catalytic cavity

**Figure 6:**
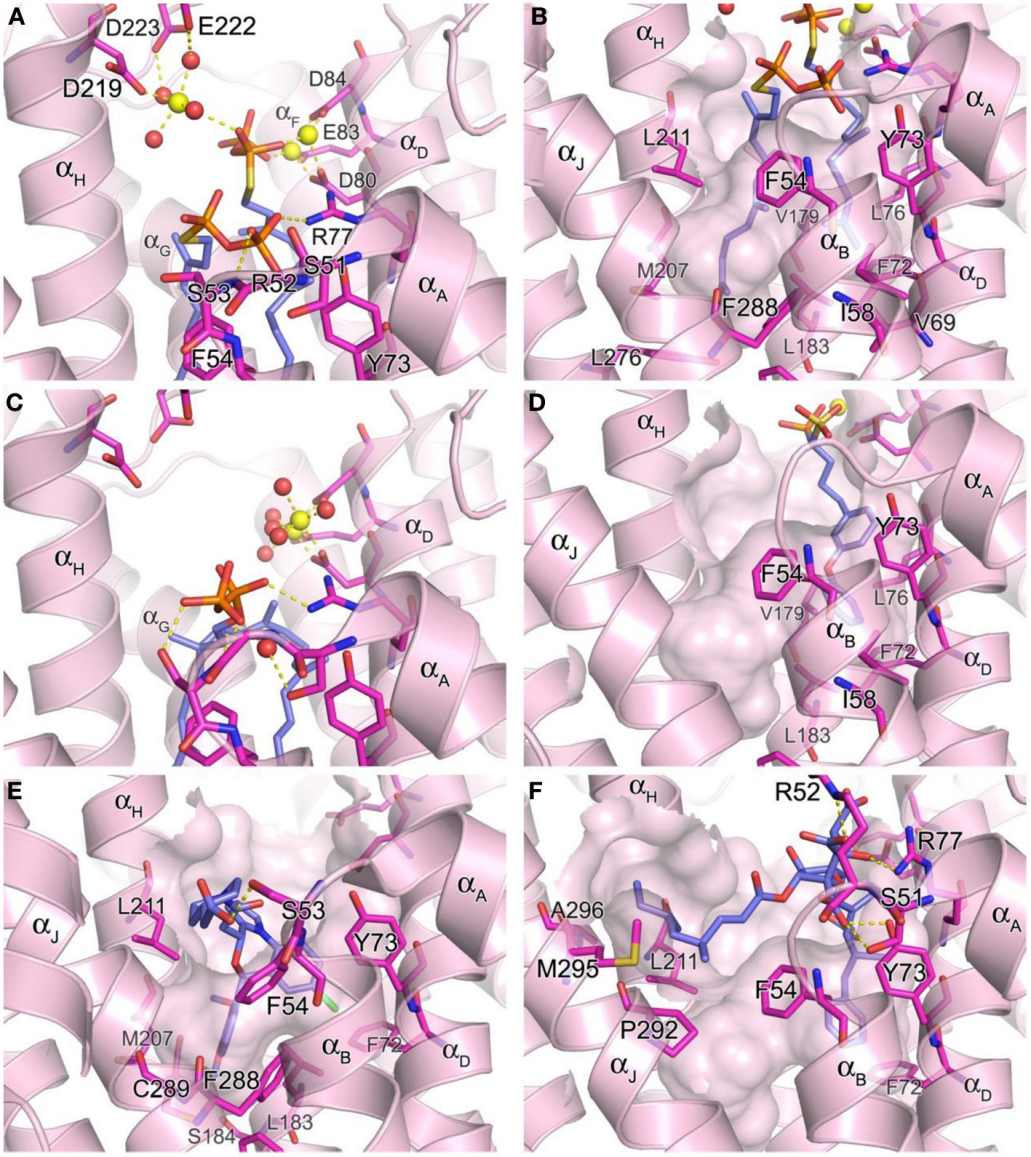
**Structure of human squalene synthase. (A,B)** Binding of the substrate mimic farnesyl thiopyrophosphate to hSQS (PDB ID: 3WEG). The Arg52 side chain is omitted for clear view of the two pyrophosphate groups in **(A)**. The large hydrophobic cavity that accommodates the substrate tails is represented in **(B)**. **(C)** Binding of the reaction intermediate PSPP shown in the same orientation as in **(A)** (PDB ID: 3WEH). For clarity of the view, residues (redundant with those in **A**) are not labeled. **(D)** Binding of a bisphosphonate inhibitor, compound **27** (PDB ID: 3LEE). **(E)** Binding of a benzoxazepine inhibitor, compound **49** (PDB ID: 3V66). **(F)** Binding of zaragozic acid A (**38**) (PDB ID: 3VJC).

The hSQS catalysis proceeds in a two-step reaction where the reductive “head to head” condensation of two FPP molecules first produces a stable cyclopropylcarbinyl diphosphate intermediate (Figure 2), the presqualene pyrophosphate (PSPP), which then undergoes heterolytic isomerization, and reduction by NADPH to give squalene (Figure 2). Binding of the two substrate molecules and the reaction intermediate are best characterized with the recently released crystal structures by Liu and co-workers (PDB IDs: 3WEG, 3WEH, 3WEI, 3WEJ, and 3WEK). The pyrophosphate of the donor FPP molecule, which yields the allylic carbocation for the first half reaction, binds in between the two DXXED motifs with three Mg^2+^ ions mediating the interaction in a pattern similar to those described for hFPPS and hGGPPS earlier (Figure 6A). The pyrophosphate of the acceptor FPP, on the other hand, binds near the flexible loop connecting α_A_ and α_B_ (i.e., Ser51-Phe54), often referred to as the “flap,” via direct interactions with residues of the loop as well as nearby residues, Tyr73 and Arg77 (Figure 6A). The isoprenyl tails of the both FPP molecules extend into a large, deep hydrophobic pocket in the center of the protein (Figure 6B). The residues Ile58, Val69, Phe72, Tyr73, Lue76, Arg77, Val175, and Lue183 provide hydrophobic contacts for the donor FPP tail on one side, and Ala176, Phe187, Met207, Leu211, and Tyr276, for the acceptor FPP tail on the other side. The residues Phe54, Val179, Lue183, and Phe288, lining the middle of the pocket, interact with the both FPP tails. The tails also make tight van der Waals contact with each other, which likely further stabilizes their binding to the enzyme. After the first half reaction, the intermediate formed (i.e., PSPP) stays bound at the same site although the conformation of the α_A_ α_B_ flap changes slightly (Figure 6C vs. Figure 6A).

To date, the binding of NADPH to hSQS has not been fully characterized (crystallographically) and there is no sequence found in this enzyme that is similar to the known pyridine nucleotide binding motifs. However, the protein depression formed by the residues Phe54, Lue211, Pro292, Met295, and Ala296 has been proposed as the cofactor binding site based on its location, as well as the conservation of these residues across all species (Liu et al., 2012). In support of this notion, this cavity is not conserved in related enzymes that do not require the nucleotide cofactor, such as dehydrosqualene synthase. The adjacent flexible region composed of the α_K_ and the following loop may also be involved in the binding of NADPH and/or function as a lid that regulates the access of the cofactor to its binding site.

### Inhibitor binding

Owing to the nature of its catalytic activity and the structures of its substrate, intermediate and product (Figure 2), hSQS has a very large active site cavity with various binding motifs for multiple ligands. Consequently, a number of structurally diverse classes of hSQS inhibitors have been reported. Some of the inhibitors have been co-crystallized with the target enzyme, and the crystal structures clearly demonstrate different binding modes of these compounds. For example, compound **27** (BPH-652), a representative of a substrate analog, binds to the donor FPP binding site in the same manner as the natural substrate FPP (PDB ID: 3LEE) (Figure 6D). The inhibitor's phosphonosulfonate group binds to the DXXED motifs, and its diphenylether side chain occupies one side of the substrate tail binding pocket, making close hydrophobic contacts with the aromatic and alkyl side chains of the residues Phe54, Ile58, Val69, Phe72, Tyr73, Lue76, Val179, Leu183, and Phe288 (Figure 6D). In contrast, benzoxazepine-based compounds, such as **47** and **48**, bind to hSQS through interactions with the substrate tail binding pocket and with the α_A_ α_B_ flap (PDB IDs: 1EZF, 3ASX, 3Q2Z, and 3V66) (Figure 6E). The benzoxazepine scaffold sits at the base of the hydrophobic pocket, with the dimethoxyphenyl substituent occupying the deepest corner of this pocket, formed by the residues Leu183, Ser184, Phe187, Ala204, Met207, Phe288, and Cys289 (Figure 6E). These inhibitors also have a structurally varied substituent containing a (di)carboxylic acid group, which makes van der Waals interactions with the side chain of Phe54 and polar interactions with other residues of the α_A_ α_B_ flap (Figure 6E). The binding of the benzoxazepine derivatives causes significant conformational changes in the flap residues, widening the base of the active site cavity.

The binding of zaragozic acid A (**38**) to hSQS has also been characterized. As expected, the tricarboxylic acid core binds to the PSPP pyrophosphate binding site, forming extensive H-bond and electrostatic interactions with the α_A_ α_B_ flap residues as well as Tyr73 and Arg77 (Figure 6F). The 4,6-dimethyloct-2-enoic acid side chain occupies the donor FPP tail binding site, with interactions similar to those described for the side chain of the substrate analog **27**, whereas the other side chain extends into the presumed NADPH binding site, making hydrophobic contacts with the residues Phe54, Lue211, Pro292, Met295, and Ala296 (Liu et al., 2012).

### Future directions in drug discovery targeting the human prenyl synthase enzymes FPPS, GGPPS, and SQS

The human enzymes FPPS, GGPPS, and SQS occupy strategic posts in modulating the levels of both down-stream and up-stream metabolites derived from the mevalonate pathway. These metabolites play an essential role in cell viability, signaling and proliferation, and affect a plethora of biochemical events that may impact human diseases from atherosclerosis to dementia. An important concern about inhibiting targets with such a large range of biochemical effects may be the potential of serious *in vivo* toxicities. This question can *never* be answered without the discovery of selective inhibitors that allow efficient *in vivo* investigations in animal models and clinical studies. At this time, the clinical benefit of chronic inhibition of HMG-CoA reductase for the treatment of hypercholesterolemia is well established. Therefore, a therapeutic window has been established between inhibiting the first control step of the entire mevalonate pathway (i.e., inhibiting HMC-CoA reductase) and reducing sufficiently the levels of all metabolites, including cholesterol to achieve clinical benefits for patient at high risk for cardiovascular diseases. Presumably, from a mechanistic point of view, hSQS inhibitors can be as safe and effective as HMG-CoA inhibitors in lowering cholesterol. Similarly, inhibition of hFPPS (and consequently osteoclastic activity) with compounds that target the bone tissue and have limited systemic exposure is an established way of treating lytic bone disease. However, it is still unknown whether effective treatment of non-skeletal cancers can be achieved with hFPPS and/or hGGPPS inhibitors. A clear causative relationship has yet to be established between up-regulation of isoprenoid biosynthesis and the progression (or initiation?) of neurodegeneration in the Alzheimer's diseases. Such important biomedical questions can never be answered without the discovery of molecular tools that specifically inhibit these prenyl synthase enzymes *in vivo* and exhibit good exposure in non-skeletal tissues, as well as permeability through the blood-brain barrier.

### Conflict of interest statement

The authors declare that the research was conducted in the absence of any commercial or financial relationships that could be construed as a potential conflict of interest.
